# The Treatment of War Wounds

**Published:** 1918-03

**Authors:** 


***The Treatment of War Wounds.*** A discussion of the Aseptic and Open-air Treatment as Compared with the Antiseptic Treatment. By FREDERIC A. BESLEY, Major, M. R. C., U. S., Army Base Hospital No. 12, with B. E. F. Abs. from *Surgery, Gynecology, and Obstetrics,* Jan. 1918.

B. states that although he was prejudiced against the widespread use of antiseptics, he attempted to give them a fair trial in war surgery. He refers especially to the Carrel-Dakin treatment and the use of " b.i.p. " (a preparation composed of 50 o/o iodoform, 25 o/o bismuth subnitrate and 25 o/o liquid paraffin).

Assuming that every wound is potentially infected, the author believes that any logical treatment should begin by maintaining and strengthening the defensive forces of the body, and then provide for the destruction or removal of the invading organisms. This achievement is not easy in practice, because at present there is not, the author believes, any germicide that can be applied to living tissue which will destroy bacteria deep in the lymphatic spaces without materially lowering the resistance of the tissue.

The nutrition of the cell is the principal factor in increasing its resistance. Although pus on the surface does little harm, that lying deeper may not only absorb and distribute toxins, but tends to shut off the blood supply by creating a pressure.

If wounds are treated within the first few hours by thorough cleansing and excision of devitalized tissues primary suture almost always results in healing by first intention. B. believes, however, that in deep thigh wounds, sutures should be inserted but not tied within forty-eight hours.

The author is strongly opposed to the packing of wounds with gauze, but thinks that soft rubber tissue may be used to advantage. He believes that " b. i. p. " is useless as a disinfectant, and that it is detrimental from other viewpoints. Out of a hundred cases treated by " b. i. p. ", 28 showed evidence of bismuth poisoning, 19 had hemorrhagic nephritis.

In cases of serious infection from wounds of long standing the author believes that complete drainage is the first essential. This should be secured by wide incisions both horizontal and longitudinal. He believes that all devitalized tissue and foreign substances should be removed, but he protests against the removal of very much of the surrounding tissues where " Nature has thrown up a barrier... which had better not be disturbed during the stage in which we are attempting to secure perfect drainage ".

Even in such cases the author urges the avoidance of tubes, gauze, rubber tissues, etc. Rubber tissue and paraffin gauze, however, may be used for the purpose of preserving a flap.

When complete drainage is obtained the author doubts that irrigating solutions are of any value. Hot baths and hot moist dressings, however, may tend to promote nutrition, and thereby strengthen the defences of the body. After applying the hot baths or hot packs for a period of about twenty-four hours the author has found exposure to the open air with no dressing whatever for twenty out of twenty-four hours to be the best method of procedure. During two intervening periods of two hours each, hot packs or baths should be used. An electric light introduced under the protecting screen may serve to keep the exposed region heated.

When secondary suture is practised on such cases in order to shorten convalescence, the Carrel-Dakin solution may play an important rôle. If it is carefully applied it will secure a granulating surface free from bacterial infection. When smears and cultures show the surfaces to be aseptic, many such wounds may be sutured successfully. If the Carrel-Dakin method is to be used, services should be equipped with the proper facilities and personnel to make its application safe.

